# Knowledge, Perceptions, and Readiness of Telepharmacy among Hospital Pharmacists in Saudi Arabia

**DOI:** 10.3390/healthcare11081087

**Published:** 2023-04-11

**Authors:** Nehad J. Ahmed, Ziyad S. Almalki, Asmaa H. Alsawadi, Abdulmohsen A. Alturki, Abdulaziz H. Bakarman, Alwaleed M. Almuaddi, Saeed M. Alshahrani, Meshari B. Alanazi, Ahmed M. Alshehri, Ahmed A. Albassam, Abdullah K. Alahmari, Ghada M. Alem, Saad A. Aldosari, Ahmad A. Alamer

**Affiliations:** 1 Department of Clinical Pharmacy, College of Pharmacy, Prince Sattam bin Abdulaziz University, Al Kharj 11942, Saudi Arabia; 2 Department of Pharmacy Practice and Pharmaceutical Sciences, College of Pharmacy, Almaarefa University, Riyadh 13713, Saudi Arabia

**Keywords:** hospital pharmacists, knowledge, perception, readiness, telepharmacy

## Abstract

Telepharmacy is a technology-based service that provides promoted services such as counseling, medication administration and compounding, drug therapy monitoring, and prescription review. It is unclear whether hospital pharmacists possess the necessary knowledge, attitudes, and willingness to practice telepharmacy. The current study sought to investigate Saudi Arabian hospital pharmacists’ understanding, attitudes, and level of preparedness for telepharmacy services. A total of 411 pharmacists responded to the survey. Only 43.33% of the respondents agreed that telepharmacy is available in Saudi Arabia and 36.67% of the respondents agreed that patients in rural areas can have more medication access and information via telepharmacy. Only 29.33% of pharmacists agreed that telepharmacy improves patient medication adherence, and about 34.00% of the pharmacists agreed that telepharmacy saves patients money and time by eliminating the need for them to travel to healthcare facilities. This research found that hospital pharmacists were unsure of their level of knowledge, their attitude toward telepharmacy, and their willingness to incorporate it into their future pharmacy practices. To ensure that tomorrow’s pharmacists have the skills they need to provide telepharmacy services, telepharmacy practice models must be incorporated into the educational programs that prepare them.

## 1. Introduction

Using information and communication technology in pharmacy and healthcare opens up new ways to provide health services and helps solve the problem of a lack of health professionals [1]. Electronic health records and other new technologies, such as electronic health information systems, make it easier for pharmacists to find information about an exam or a drug therapy that was prescribed. By using these technologies, pharmacists can improve how drugs are used and also get the word out about telepharmaceutical services [2]. 

The National Association of Boards of Pharmacy defines it as “the provision of pharmacological treatment to patients at a distance using information and communication technology” [3]. Telepharmacy is a technology-based service that offers services such as prescription evaluation, medicine administration and compounding, drug therapy monitoring, and counseling [4,5,6]. Telepharmacy services include reviewing pharmaceutical orders, giving out drugs, counseling and evaluating patients, monitoring therapeutic drug use, and managing medication treatment [7].

Telepharmacy lets patients obtain their prescriptions and other pharmaceutical care services without having to go to a pharmacy [3,4]. It was found to reduce travel costs and save time, both of which are important barriers for patients in rural and faraway areas who need healthcare services, especially the disabled and elderly [4,8,9]. Telepharmacy also helps patients stay on their medicines by making them happier and more confident in the services they receive [10,11]. In addition, it has made pharmacists’ clinical roles more effective by giving them enough time for drug counseling in a more private setting [12,13].

The social distance between patients and healthcare providers has been enabled in many countries through the facilitation of remote pharmaceutical care services such as Internet services, virtual medical consultations, e-prescriptions, and home drug delivery [14]. With its origins in addressing healthcare access issues in rural regions, telepharmacy reaches its maximum use during global health crises [6]. Telepharmacy was quickly recognized as a tool capable of overcoming many of the pandemic’s challenges. The introduction of COVID-19 has accelerated the changes that would make telepharmacy a viable option. As healthcare providers and patients recognize the benefits of telepharmacy, there is a chance that it will continue even after the pandemic is over [15]. Ibrahim et al. reported that pharmacies with remote pharmacy services could help more patients with COVID-19 and confirm COVID-19 diagnosis more quickly than pharmacies without remote pharmacy services. They also reported that pharmacies with remote services were less likely than other pharmacies to make mistakes during medication dispensing [16]. Casey et al. found that the number of mistakes made when giving out medications decreased after a telepharmacy service was put in place [3].

There are several studies about the knowledge, perceptions, and readiness of pharmacists in community pharmacies in Saudi Arabia about telepharmacy. There are also studies about the knowledge, perception, and willingness of the public to use telepharmacy services. Nonetheless, there are no studies on the knowledge, perceptions, and readiness of hospital pharmacists about telepharmacy in Saudi Arabia, except in one conference abstract [17]. So, the present study included pharmacists who are working in hospital pharmacies. Therefore, the goal of this study was to find out what Saudi Arabian hospital pharmacists know, how they feel about telepharmacy services, and how ready they are to use them.

## 2. Materials and Methods

Between March and May 2020, a cross-sectional study was conducted among Saudi Arabian hospital pharmacists to assess their knowledge, perceptions, and readiness for telepharmacy. Pharmacists who worked in Saudi Arabian hospital pharmacies were included in the study. Pharmacists who worked in other settings such as community pharmacies and pharmaceutical companies were not included in the study. The subjects’ involvement was entirely voluntary, and there was no monetary incentive.

An online survey was used to obtain data from the participants, and it was based on a validated questionnaire used by a previous study [18]. The questionnaire was provided in English. After taking into account their experience and knowledge of the study subject, four pharmacy academicians reviewed the content validity of the questionnaire. After that, we sent the questionnaire to a few pharmacists as a pilot test to weed out any unnecessary or inadequate queries (the questionnaire was attached as a supplementary file).

The questionnaire was created using Google Forms as an online form. Data were collected on respondent demographics, employment statistics, hospital pharmacist knowledge about telepharmacy, pharmacist telepharmacy attitudes, and pharmacist telepharmacy preparedness. The online survey was sent to the pharmacists’ emails, and it was sent to other pharmacists using WhatsApp. After the pharmacists sent their responses, we checked the surveys to ensure that the surveys were filled out completely.

The sample size was calculated using the RAO sample size calculator, and using a margin error of 5%, confidence level of 95%, and response distribution of 50%. The minimum recommended size of our survey was 377 pharmacists.

The data were gathered using an Excel spreadsheet, and the descriptive results were presented in the form of numbers and percentages. After that, the Statistical Package for Social Science (SPSS) was used for the data analysis. Differences in pharmacists’ knowledge, perception, and readiness scores in relation to years of experience were assessed using an independent samples *t*-test. The level of significance was set at *p* < 0.05. The study was approved by the Research Ethics Committee/Health and Science Disciplines at Prince Sattam Bin Abdulaziz University with an approval number REC-HSD-134-2022.

## 3. Results

The survey was sent to five hundred pharmacists. A total of four hundred eleven individuals responded to the survey (the response rate was 82.2%). Males made up 54.01% of the respondents, with 71.29% of them being between the ages of 30 and 39 years, and the age of 24.33% of them was between 20 and 29 years. Most of the respondents had a bachelor’s degree in pharmacy (46.71%) or a PharmD degree (27.01%), and 20.44% of them had a master’s degree. Most of the responders (87.10%) received their most recent pharmacy degree or training in Saudi Arabia, and 5.11% of them received a pharmacy degree or training in the United States (Table 1).

**Figure 1:**
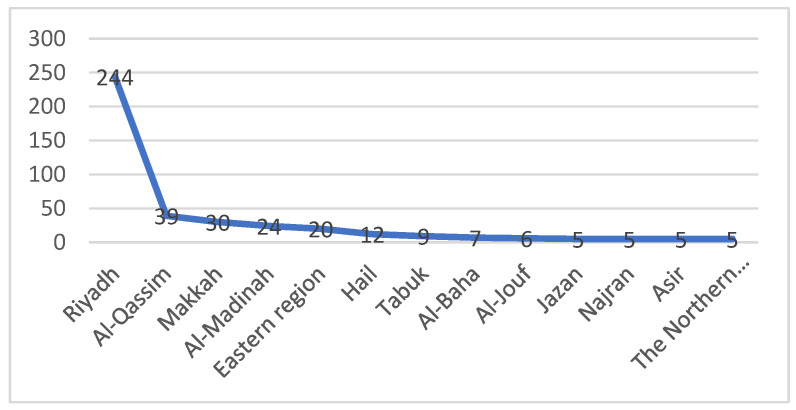
The regions where pharmacists work.

Approximately 59.37% of pharmacists were employed in the Riyadh region, 9.49% in the Al-Qassim region, and 7.30% in the Makkah region (Figure 1).

Most of the pharmacists (62.53%) worked in urban areas, and 51.34% of them had less than five years of experience in hospital pharmacies. About 48.66% of the pharmacists were staff pharmacists, about 28.96% of them worked in an outpatient pharmacy, and about 44.28% of them worked in a primary healthcare institution. Most of the respondents had never provided pharmaceutical services via telepharmacy before (63.50%). More than half of the respondents (54.01%) stated that social media was their primary source of information, while 33.09% said that the Ministry of Health website was their primary source of information (Table 2).

Only 43.33% of the respondents agreed that telepharmacy is available in Saudi Arabia, 44.00% of them agreed that telepharmacy played a significant role during the global COVID-19 outbreak, 38.00% of the pharmacists agreed that telepharmacy provides better counseling in terms of privacy and the length of the session, and 38.67% of them agreed that telepharmacy solves the waiting time problem in most general hospitals.

About 36.67% of the hospital pharmacists agreed that telepharmacy is involved in adverse drug reaction monitoring and reporting, 36.67% of the respondents agreed that patients in rural areas can have more medication access and information via telepharmacy, and 39.33% of them agreed that telepharmacy services can extend hospital pharmacy services outside office hours that do not offer round-the-clock pharmacy services (Table 3).

Only 29.33% of hospital pharmacists agreed that telepharmacy improves patient medication adherence, 36.66% agreed that it improves patient access to medications in rural areas, and only 26.66% of them agreed that telepharmacy has a higher error rate for medication dispensing and filling compared to the traditional pharmacy. Furthermore, 34.00% of them said that telepharmacy increases the pharmacist’s workload and commitment, about 34.00% of the pharmacists agreed that telepharmacy saves patients money and time by eliminating the need for them to travel to healthcare facilities, and about 32.00% of them agreed that they are willing to share their personal information on the online database when using telepharmacy services.

About 33.33% of hospital pharmacists said that telepharmacy minimizes the cost to establish a pharmaceutical business in comparison to a regular pharmacy. About 33.33% of the hospital pharmacists agreed that patient consultation via telepharmacy is effective, and about 34.67% of them agreed that therapeutic drug monitoring via telepharmacy in rural areas is easily completed. Furthermore, 36.67% of pharmacists said that pharmacy schools should incorporate computer sciences and telepharmacy education programs to help with future telepharmacy use, and 33.33% of them agreed that telepharmacy helps to reduce pharmacist shortages (Table 4).

About 27.74% of the hospital pharmacists agreed that they are ready to work on telepharmacy projects in rural areas, even without an incentive, and 34.55% of them said that they are ready to work after office hours if needed. About 36.5% of pharmacists stated they are ready to provide pharmaceutical advice via two-way video consultation, such as phone calls, text messaging, or voice conversations using mobile devices. More than 39% of the pharmacists said that they are ready to teach patients how to use their drug delivery device through video consultation, and more than 37% of them agreed that they are ready to undergo training in ethics and legal issues related to telepharmacy.

About 35.28% of pharmacists said that they are prepared to tackle telepharmacy implementation in all clinical settings. Furthermore, about 37.96% of pharmacists stated that they are ready to utilize telepharmacy to increase patient safety and prevent prescription errors, and 39.17% of them said that they are ready to use telepharmacy to conduct medication reconciliation. More than 36% of the pharmacists agreed that they are ready to perform remote prescription-checking using an automated medication dispensing cabinet, and 36.98% of them said that they are ready to use applications and the Internet to receive refill orders and transfer prescriptions (see Table 5).

The differences in pharmacists’ knowledge, perception, and readiness scores in relation to years of experience are shown in Table 6. Regarding the knowledge, the correct answers were given 1 and the incorrect answers were given 0, and after that we calculated the average knowledge score. Regarding perceptions and readiness, strongly agree and agree were given 1; neutral, disagree, and strongly disagree were given 0; after that, we calculated the average perceptions and readiness scores. The average knowledge score, perceptions score, and readiness score were higher in pharmacists who had less than 5 years compared with pharmacists who had 5 or more years of experience (*p* value less than 0.05).

## 4. Discussion

In this study, hospital pharmacists in Saudi Arabia were asked about their knowledge, perceptions, and preparation for telepharmacy. Less than half of the pharmacists said that telepharmacy is available in Saudi Arabia. In general, there was uncertainty and variation in the response of the hospital pharmacists regarding their knowledge, which could be explained by the fact that it has not yet been fully developed and made widely available. Similarly, Elnaem et al. noted in their study that there was some uncertainty in the responses of future pharmacists in Malaysia [18]. Omran et al. reported that approximately 40% of Egyptian pharmacists were unfamiliar with the term telepharmacy [19]. Moreover, Tegegne et al. reported that only 32.4% of the pharmacy students in northwest Ethiopia had good knowledge of telepharmacy [20].

About 44% of pharmacists reported that telepharmacy played a big role during the COVID-19 outbreak around the world. Telepharmacy could overcome many of the challenges presented by the COVID-19 pandemic while still providing quality patient care [15]. Elnaem et al. stated that 93.8% of future Malaysian pharmacists agreed that telepharmacy played a big role during the COVID-19 outbreak around the world [18]. Unni et al. stated that the introduction of COVID-19 has hastened the changes required to make telepharmacy a viable option. They also stated that telepharmacy may continue even after the pandemic is over, as healthcare providers and patients recognize its benefits [15]. According to Ibrahim et al. pharmacies with remote pharmacy services could assist COVID-19 patients faster than pharmacies without remote pharmacy services [16]. Telepharmacy is a remote pharmaceutical care procedure. It has been used worldwide during the COVID-19 pandemic, with the aim of preserving the health of patients and professionals [21]. Dat et al. reported that about 87% of pharmacists have used telepharmacy in their pharmacy practice in response to the COVID-19 pandemic in Ho Chi Minh City, Vietnam. They also reported that the provision of medical information and remote medication counseling was an urgent need in the context of limited travel because of the COVID-19 pandemic [22].

Pharmacists are medical experts in charge of making sure that patients have access to the medications they require. Pharmacists must develop methods for the purchase, storage, and distribution of medications in order to accomplish this. This is in line with the pharmacist’s responsibility for addressing sufficient access to medications, one of the social determinants of health. When pharmacists provide care to a community that is underprivileged, rural, or remote, this duty becomes vital [23]. More than one-third of pharmacists in the current study said that telepharmacy can provide more drug availability and information to patients in remote places, and about half were uncertain. Ibrahim et al. [16] reported that many healthcare practitioners use telepharmacy services to improve patients’ access to pharmaceutical care. Furthermore, the Elnaem et al. study and the Poudel and Nissen study [8,18] showed that telepharmacy plays a significant role in improving drug access for patients in rural areas. Pathak et al. stated that telepharmacies are a suitable solution for expanding medication access and that using a telepharmacy would not have a negative impact on medication quality [24]. Omboni et al. reported that telepharmacy can provide access to healthcare services in remote communities [25].

When feasible, telepharmacy helps patients avoid waiting in a clinic with other sick patients, save time traveling, avoid work loss, and get themselves and their families healthy. Telepharmacy saves patients money and time by eliminating the need for them to visit healthcare facilities, according to 34.00% of pharmacists in the current study. Previous research has demonstrated that telepharmacy reduces travel costs and saves time, which are considered a major barrier to patients in rural and distant settings, particularly the disabled and elderly, receiving healthcare services [4,9]. More than 91% of pharmacists in Alanazi et al.’s study considered that employing a telepharmacy system may save them time and money [17]. The beneficial effect of telepharmacy in conserving patients’ resources was evaluated positively by study participants, with 91 percent agreement [18], according to Elnaem et al.

It is more difficult for people to fill prescriptions and obtain access to other essential services in rural regions due to a lack of pharmacies. Rural areas with a shortage of pharmacists experienced a reduction in turnaround time for clinical pharmacy services and an increase in medication errors linked to pharmacies. According to the current survey, about 33.33% of pharmacists believed that telepharmacy helps to minimize pharmacist shortages. Telepharmacy has already been shown to help with the provision and delivery of healthcare services for patients in rural locations or in situations when access to healthcare or pharmaceutical services is problematic for any reason [26,27,28]. In rural areas, where there is a shortage of pharmacists and clinicians, telepharmacy and telehealth clinical pharmacy services can fill the gap. Telepharmacy is a solution to the shortage of pharmacy employees, according to Baldoni et al. in which pharmaceutical services are provided remotely [29]. Furthermore, 75% of respondents in Elnaem et al.’s study agreed that telepharmacy may help alleviate the current pharmacist shortage [18]. Poudel and Nissen reported that telepharmacy addresses pharmacist shortages in rural areas and improves patient access to pharmaceuticals and pharmacy services [8].

The current study showed lower telepharmacy readiness among participants. Elnaem et al. reported that about 67% of the senior pharmacy students in a Malaysian public pharmacy school had high knowledge, and 68% of them showed high readiness levels. They also reported that factors such as a lack of incentive and an excessive workload were linked to participants’ telepharmacy preparedness [18]. Payment and reimbursement issues, as well as a lack of access to information technology infrastructure, were among the most important roadblocks, according to Ameri et al. [30]. According to Omran et al. the main barriers to telepharmacy practice included a lack of professional training, ethical concerns, and a formal practice framework [19]. Dat et al. reported that about 87.2% of the pharmacists in their study were ready to use telepharmacy [22]. Pharmacy colleges should incorporate telepharmacy practice models into their curricula in order to better prepare future pharmacists to provide telepharmacy services. Additionally, pharmacists’ knowledge and preparedness to utilize telepharmacy can be improved through the delivery of lectures, workshops, and conference attendance.

The present study showed that the average knowledge score, perceptions score, and readiness score were higher in pharmacists who had less than 5 years compared with pharmacists who had 5 or more years of experience. According to Fortnez et al.’s study, only younger pharmacist participants had higher levels of interest in and favorable views toward using telemedicine resources [31]. Ng and Sze reported that community pharmacists who are younger and have fewer years of experience in the workforce are more likely to have a favorable outlook toward the adoption of telepharmacy [32]. Telepharmacy services would appeal mainly to younger pharmacists, according to Ilki et al. [33]. Continuing professional education is a relevant venue to increase knowledge and promote a positive attitude towards telepharmacy, especially for older pharmacists.

The first limitation of the study was that it was based on an online survey, which could have resulted in selection bias. The study’s second limitation was that we have the contact information of several pharmacists who worked in several cities in Saudi Arabia, but we could not send emails and messages to all of the pharmacists who work in Saudi Arabia. Therefore, the present study included a small number of pharmacists, making the findings’ generalizability questionable. Further studies with larger sample sizes are needed to obtain more accurate or representative results.

## 5. Conclusions

From the results of this research, it is clear that there was uncertainty among hospital pharmacists regarding their level of telepharmacy knowledge, their attitude toward the practice, or their level of preparedness to incorporate it into their future pharmacy operations.

## Figures and Tables

**Table 1:** Demographic data of the respondents (n = 411).

Variable	Category	Number	Percentage
Gender	Male	222	54.01
	Female	189	45.99
Age	20–29	100	24.33
	30–39	293	71.29
	40–49	16	3.89
	More than 49	2	0.49
Education level	Bachelor’s degree	192	46.71
	Pharm.D.	111	27.01
	Master’s degree	84	20.44
	Doctorate degree (Ph.D.)	12	2.92
	Other	12	2.92
In which country have you earned your latest pharmacy degree or training?	Saudi Arabia	358	87.10
	UK	9	2.19
	USA	21	5.11
	Australia	6	1.46
	Malaysia	3	0.73
	Egypt	5	1.22
	India	3	0.73
	Pakistan	2	0.49
	Other	4	0.97
Nationality	Saudi Arabia	400	97.32
	Others *	11	2.68

* Others: Pharmacists who work in Saudi Arabia, but are not Saudis (For example, Pakistani, Indian, Jordanian, and Egyptian pharmacists).

**Table 2:** Employment data of the respondents (n = 411).

Variable	Category	Number	Percentage
What is your area of work?	Rural	154	37.47
	Urban	257	62.53
Years of experience in pharmacy	Less than 5 5–10 11–15 More than 15	211 162 15 23	51.34 39.41 3.65 5.60
What is your job position at your hospital pharmacy?	Staff pharmacist Clinical pharmacist Pharmacy supervisor Pharmacy manager Other	200 73 24 72 42	48.66 17.76 5.84 17.52 10.22
What is your hospital pharmacy setting?	Out-patient pharmacy In-patient pharmacy Clinical pharmacy Other	119 116 72 104	28.96 28.22 17.52 25.30
What is the level of your healthcare institution?	Primary Secondary Tertiary	182 91 138	44.28 22.14 33.58
Have you previously provided pharmaceutical services through telepharmacy?	Yes No	150 261	36.50 63.50
Source of information	Local and international channels	120	29.20
	Social media	222	54.01
	WHO website and social pages	124	30.17
	Scientific journals	104	25.30
	Ministry of Health website	136	33.09
	Colleagues	119	28.95
	Others	135	32.85

**Table 3:** Telepharmacy knowledge among pharmacists (n = 150).

Item	Response	Number	Percentage
Telepharmacy is available in Saudi Arabia.	Yes	65	43.33
	No	13	8.67
	Don’t know	72	48.00
Information communication technology (ICT) knowledge is important for pharmacists in how to conduct telepharmacy.	Yes	67	44.67
	No	15	10.00
	Don’t know	68	45.33
Telepharmacy played a big role during the COVID-19 outbreak around the world.	Yes	66	44.00
	No	15	10.00
	Don’t know	69	46.00
Telepharmacy does require a strong Internet connection or high-performance technology.	Yes	61	40.67
	No	17	11.33
	Don’t know	72	48.00
Telepharmacy provides better counseling in terms of privacy and length of the session.	Yes No Don’t know	57 16 77	38.00 10.67 51.33
Telepharmacy solves the waiting time problem in most general hospitals.	Yes No Don’t know	58 20 72	38.67 13.33 48.00
Telepharmacy is also involved in adverse drug reaction monitoring and reporting.	Yes No Don’t know	55 20 75	36.67 13.33 50.00
Telepharmacy is conducted by drug information services during office hours and by emergency departments after office hours.	Yes No Don’t know	55 18 77	36.67 12.00 51.33
Patients from rural areas can have more medication access and information via telepharmacy.	Yes No Don’t know	55 21 74	36.67 14.00 49.33
Telepharmacy services can extend hospital pharmacy services outside office hours that do not offer round-the-clock pharmacy services.	Yes No Don’t know	59 17 74	39.33 11.33 49.33

**Table 4:** Telepharmacy perceptions among pharmacists (n = 150).

Items	Strongly Disagree	Disagree	Unsure	Agree	Strongly Agree
Telepharmacy improves patient’s adherence to the medication.	9 (6.00)	8 (5.33)	89 (59.33)	35 (23.33)	9 (6.00)
Telepharmacy has a higher error rate for medication dispensing and filling compared to traditional pharmacy.	5 (3.33)	14 (9.33)	91 (60.67)	32 (21.33)	8 (5.33)
Telepharmacy enhances patient’s access to medications in rural areas.	4 (2.67)	7 (4.67)	84 (56.00)	44 (29.33)	11 (7.33)
Telepharmacy provides a complete privacy setting during the consultation period.	3 (2.00)	10 (6.67)	86 (57.33)	40 (26.67)	11 (7.33)
Telepharmacy increases pharmacist’s workload and commitment.	3 (2.00)	9 (6.00)	87 (58.00)	37 (24.67)	14 (9.33)
Telepharmacy helps patients save their money and travel time to reach the healthcare facilities.	4 (2.67)	6 (4.00)	89 (59.33)	35 (23.33)	16 (10.67)
I am willing to share my personal information on the online database when using telepharmacy services.	5 (3.33)	11 (7.33)	86 (57.33)	37 (24.67)	11 (7.33)
Telepharmacy minimizes the cost to establish a pharmaceutical business in comparison to the regular pharmacy.	5 (3.33)	9 (6.00)	86 (57.33)	42 (28.00)	8 (5.33)
Patient consultation via telepharmacy is effective.	3 (2.00)	10 (6.67)	87 (58.00)	41 (27.33)	9 (6.00)
Pharmacy schools should provide education programs on IT and telepharmacy to assist in the future utilization of telepharmacy.	4 (2.67)	7 (4.66)	84 (56.00)	40 (26.67)	15 (10.00)
Therapeutic drug monitoring via telepharmacy in rural areas is easily monitored.	4 (2.67)	11 (7.33)	83 (55.33)	40 (26.67)	12 (8.00)
Security is a greater concern in a remote site telepharmacy than in a traditional community pharmacy.	4 (2.67)	7 (4.66)	81 (54.00)	42 (28.00)	16 (10.67)
Telepharmacy helps to minimize the shortage of pharmacists.	4 (2.67)	8 (5.33)	88 (58.67)	38 (25.33)	12 (8.00)

**Table 5:** Telepharmacy readiness among pharmacists (n = 411).

Items	Strongly Disagree	Disagree	Unsure	Agree	Strongly Agree
I am ready to work on telepharmacy projects in rural areas, even without an incentive.	19 (4.62)	37 (9.00)	241 (58.64)	87 (21.17)	27 (6.57)
I am ready to work after office hours if needed.	15 (3.65)	20 (4.87)	234 (56.93)	108 (26.28)	34 (8.27)
I am ready to conduct drug counseling via two-way video consultation. *	10 (2.43)	20 (4.87)	231 (56.20)	120 (29.20)	30 (7.30)
I am ready to teach patients how to use their drug delivery devices through video consultation.	8 (1.95)	25 (6.08)	217 (52.80)	125 (30.41)	36 (8.76)
I am ready to undergo training in ethics and legal issues related to telepharmacy.	8 (1.95)	18 (4.38)	231 (56.20)	113 (27.49)	41 (9.98)
I am ready to face the implementation of telepharmacy in all healthcare settings.	8 (1.95)	22 (5.35)	236 (57.42)	106 (25.79)	39 (9.49)
I am ready to conduct a home medication review (HMR) through telepharmacy.	8 (1.95)	22 (5.35)	239 (58.15)	112 (27.25)	30 (7.30)
I am ready to reduce the risk of medication errors among patients through telepharmacy.	10 (2.43)	17 (4.14)	228 (55.47)	126 (30.66)	30 (7.30)
I am ready to carry the increment of workload when conducting telepharmacy.	10 (2.43)	27 (6.57)	223 (54.25)	109 (26.52)	42 (10.22)
I am ready to conduct medication reconciliation via telepharmacy services.	9 (2.19)	17 (4.14)	224 (54.50)	119 (28.95)	42 (10.22)
I am ready to perform remote prescription-checking using an automated medication dispensing cabinet.	8 (1.95)	21 (5.11)	231 (56.20)	115 (27.98)	36 (8.76)
I am ready to use applications and the Internet to receive refill orders and transfer prescriptions.	12 (2.92)	16 (3.89)	231 (56.20)	113 (27.49)	39 (9.49)

* Two-way video consultation such as telephone calls, text messages, or voice calls through mobile applications.

**Table 6:** Differences in pharmacists’ knowledge, perception, and readiness scores in relation to years of experience.

Years of Experience	Average Knowledge Score	*p* Value
Less than 5	0.42	0.02
More than 5 years	0.38	
**Years of experience**	**The average score for perceptions**	***p* value**
Less than 5 More than 5 years	0.39 0.28	0.003
**Years of experience**	**The average score for readiness**	***p* value**
Less than 5 More than 5 years	0.41 0.31	0.008

## Data Availability

Data available on request due to restrictions.

## Supplementary Materials

The following supporting information can be downloaded at: https://www.mdpi.com/article/10.3390/healthcare11081087/s1, File S1: the questionnaire.
