# Supplementary material for: Knowledge, Perceptions, and Readiness of Telepharmacy among Hospital Pharmacists in Saudi Arabia
Source: Healthcare (Basel). 2023 Apr 11;11(8):1087. doi: 10.3390/healthcare11081087 (PMC10137432; doi:10.3390/healthcare11081087)
Supplement: Supplementary file 1 [file healthcare-11-01087-s001.zip › healthcare-2277389-supplementary.pdf]

Knowledge, Perceptions, and Readiness of Telepharmacy among Pharmacists

Part A: Personal Data

Here, we would like to ask you some questions about yourself.

1. **What is your Gender?**

☐ Male ☐ Female

2. **What is your marital status?**

☐ Married ☐ Divorced ☐ Separated ☐ Widowed ☐ Never Married

3. **What is your year of birth? ________**

4. **In which region are you currently working?**

☐Riyadh ☐Makkah ☐Al-Madinah ☐Al-Qassim ☐Eastern region

☐Asir ☐Tabuk ☐Hail ☐Jazan ☐Najran

☐Al-Baha ☐Al-Jouf ☐The Northern Border region

5. **What is your area of Work?**

☐ Rural ☐ Urban

6. **How much is your total monthly income?**

☐ ≤5,000 ☐ 5,001 – 10,000 ☐ 10,001 – 15,000 ☐ 15,001 – 20,000

☐ 20,001 – 25,000 ☐ 25,001 – 30,000 ☐ ≥30,000

7. **What is your most recent pharmacy education degree?**

☐ Bachelor degree ☐ Pharm.D. ☐ Master's degree

☐ Philosophy of Doctorate (Ph.D.) ☐ Other, Please Specify ________________

8. **Years of experience in pharmacy? _______** years

9. What is your nationality?

☐ Saudi Arabia ☐ Other, Please Specify __________

10. In which country have you earned your latest pharmacy degree or training?

☐Saudi Arabia ☐UK ☐USA ☐Australia ☐Malaysia

☐Egypt ☐India ☐Pakistan ☐Other, please specify _________

11. What is your job position at your hospital pharmacy?

☐ Staff Pharmacist ☐ Clinical Pharmacist ☐ Pharmacy supervisor

☐ Pharmacy Manager Other, please specify _________

12. What is your hospital pharmacy setting?

☐ Out-patient pharmacy ☐ In-patient pharmacy ☐ Clinical pharmacy

Other, please specify _________________

13. What is the level of your healthcare institution?

☐ Primary ☐ Secondary ☐ Tertiary

14. On average, how many hours do you usually work per week? _______ hours

15. Have you previously provided pharmaceutical services through telepharmacy? (If No, please skip question 19)

☐ Yes ☐ No

16. How many years have you been providing pharmaceutical services through telepharmacy? _______ years

17. Source of information (you can choose more than one answer)

☐ Local channels and international channels
☐ Social media
☐ WHO website and social pages

☐ Scientific journals
☐ Ministry of Health (MOH) website
☐ Colleagues
☐ Others

Part B:
Telepharmacy knowledge among pharmacists

In this section, we would like to identify your knowledge about telepharmacy. Please answer yes or no to the following question:

ITEM	Yes	No	Don’t know
1. Telepharmacy is available in Saudi Arabia	☐	☐	☐
2. Information Communication Technology (ICT) knowledge is important for pharmacists on how to conduct telepharmacy.	☐	☐	☐
3. Telepharmacy played a big role during the COVID-19 outbreak around the world.	☐	☐	☐
4. Telepharmacy does require a strong internet connection or high-performance technology.	☐	☐	☐
5. Telepharmacy provides better counseling in terms of privacy and length of the session.	☐	☐	☐

6.	Telepharmacy solves the waiting time problem in most general hospitals.	☐	☐	☐
7.	Telepharmacy is also involved in Adverse Drug Reaction monitoring and reporting.	☐	☐	☐
8.	In general hospitals, telepharmacy is conducted by drug information service during office hours and by emergency departments after office hours.	☐	☐	☐
9.	Patients from rural areas can have more medication access and information via telepharmacy.	☐	☐	☐
10.	Telepharmacy services can extend hospital pharmacy services outside office hours that do not offer round-the-	☐	☐	☐

clock pharmacy
services.

Part C

Telepharmacy perceptions among pharmacists

In this part, we want to know your perception level regarding **utilizing telepharmacy among pharmacists**. Please check the box that corresponds with your level of agreement with the statements below.

Items	Strongly Disagree	Disagree	Unsure	Agree	Strongly Agree
1. Telepharmacy improve patient's adherence to the medication	☐	☐	☐	☐	☐
2. Telepharmacy have a higher error rate for medication dispensing and filling compared to traditional pharmacy	☐	☐	☐	☐	☐
3. Telepharmacy enhance patient's access to medications in rural areas	☐	☐	☐	☐	☐
4. Telepharmacy provide the complete privacy setting during the consultation period	☐	☐	☐	☐	☐
5. Telepharmacy increase pharmacist's workload and commitment	☐	☐	☐	☐	☐
6. Telepharmacy help patients save their money and travel time to reach the healthcare facilities	☐	☐	☐	☐	☐
7. I am willing to share my personal information on the online database when using telepharmacy services	☐	☐	☐	☐	☐
8. Telepharmacy minimize the cost to establish a pharmaceutical business in comparison to the regular pharmacy	☐	☐	☐	☐	☐
9. Patient consultation via telepharmacy is effective	☐	☐	☐	☐	☐
10. Pharmacy schools should provide education programs on computers, IT, and telepharmacy to assist in future utilization of telepharmacy	☐	☐	☐	☐	☐
11. Therapeutic drug monitoring via telepharmacy in rural areas is easily monitored	☐	☐	☐	☐	☐

12. Security is a greater concern in a remote site telepharmacy than in a traditional community pharmacy	☐	☐	☐	☐	☐
13. telepharmacy help to minimize the shortage of pharmacists	☐	☐	☐	☐	☐

Part D

Telepharmacy readiness among pharmacists

In this part, we want to know your **readiness level regarding utilizing telepharmacy among pharmacists**. Please check the box that corresponds with your level of agreement with the statements below.

Items	Strongly Disagree	Disagre e	Unsur e	Agre e	Strongly Agree
1. I am ready to work on telepharmacy projects in rural areas, even without an incentive.	☐	☐	☐	☐	☐
2. I am ready to work after office hours if needed.	☐	☐	☐	☐	☐
3. I am ready to conduct drug counseling via two-way video consultation such as telephone call, text message, or voice call through mobile applications.	☐	☐	☐	☐	☐
4. I am ready to teach patients how to use their drug delivery device (e.g., inhaler, insulin pen) properly through video consultation.	☐	☐	☐	☐	☐
5. I am ready to undergo training in ethics and legal issues related to telepharmacy	☐	☐	☐	☐	☐
6. I am ready to face the implementation of telepharmacy in all healthcare settings.	☐	☐	☐	☐	☐
7. I am ready to conduct Home Medication Review (HMR) through telepharmacy	☐	☐	☐	☐	☐
8. I am ready to improve and reduce the risk of medication errors among patients through telepharmacy.	☐	☐	☐	☐	☐
9. I am ready to carry the increment of workload when conducting telepharmacy.	☐	☐	☐	☐	☐

10. I am ready to conduct medication reconciliation via telepharmacy services.	☐	☐	☐	☐	☐
11. I am ready to perform remote prescription checking before dispensing drugs from an automated medication dispensing cabinet.	☐	☐	☐	☐	☐
12. I am ready to use mobile applications and the Internet to receive refill orders from patients and transfer prescriptions.	☐	☐	☐	☐	☐
